# A fatal case of severe neck abscess due to a third branchial cleft fistula: morphologic and immunohistochemical analyses

**DOI:** 10.1186/s13000-016-0540-0

**Published:** 2016-09-15

**Authors:** Fang Tong, Yue Liang, Muhammad Fasahat Khan, Lin Zhang, Wenhe Li, Mohammed Mahmoodurrahman, Yiwu Zhou

**Affiliations:** 1 Department of Forensic Medicine, Tongji Medical College, Huazhong University of Science and Technology, No. 13 Hangkong Road, Hankou Wuhan, 430030 People’s Republic of China; 2 College of Medicine, Alfaisal University, Riyadh, Saudi Arabia

**Keywords:** Third branchial cleft fistula, Branchial cleft anomaly, Neck abscess, Immunohistochemistry

## Abstract

**Background:**

Branchial cleft anomalies constitute a frequently encountered and commonly non-lethal disease in otolaryngology, and result from aberrant embryonic development. The third branchial cleft fistula is one of the four known specific types of branchial cleft anomalies, and always presents as recurrent neck abscess and suppurative thyroiditis. Here, we report an unexpected death due to severe neck infection following a third branchial cleft fistula.

**Case presentation:**

A 19-year-old man was sent to the hospital with a 1-week history of recurrent left-sided neck abscess, and was scheduled for incision and drainage of the abscess. However, before the surgery was performed, the man’s condition deteriorated and he died. A review of his medical history showed that he had undergone a previous incision and drainage for a neck abscess 2 years ago. Postmortem examination revealed that the fatal neck abscess was induced by a third branchial cleft fistula.

**Conclusions:**

We conclude that a histopathological examination of neck tissue combined with a detailed review of medical history and examination of ultrasonographic and CT images can provide a rapid and accurate diagnosis of third branchial cleft fistula. This common, non-lethal disease can potentially lead to death if the neck infection is not properly diagnosed and treated. In medico-legal practice, medical examiners should be aware of this condition, as this knowledge would be important in the diagnosis of the cause of death.

## Background

Branchial cleft anomalies are frequently encountered by otolaryngologists and constitute one of the uncommon anomalies of embryonic development [1]. They arise from malformations occurring during development of the fetal branchial apparatus and formation of the epithelized tract, and these, in turn, may give rise to some congenital anomalies of the head and neck region [2]. Approximately 17 % of all pediatric neck masses are related to branchial cleft anomalies [1].

The third branchial cleft fistula is a rare type of branchial cleft anomaly that only accounts for 2–8 % of all the anomalies [3, 4], with approximately 94 % of these anomalies found on the left side [5]. Anatomical findings show that the third branchial cleft fistula passes along the carotid sheath and then travels between the glossopharyngeal and hypoglossal nerves. It pierces the thyrohyoid membrane and tracks above the superior laryngeal nerve ending at the internal opening in the cephalad location of the pyriform fossa, forming a sinus fistula. Sometimes it has an external opening in the lower neck or at the anterior border of the sternocleidomastoid muscle [1, 6]. Due to the position of the common inner opening of third branchial cleft fistula [7], it is susceptible to neck abscesses [8].

Here, we present a fatal case involving a third branchial fistula followed by a severe neck abscess. To the best of our knowledge, reported cases of death due to this disease are rare. The autopsy, pathology, and diagnostic standards of third branchial fistula are discussed in this paper.

## Case presentation

### Case history

**Fig. 1:**
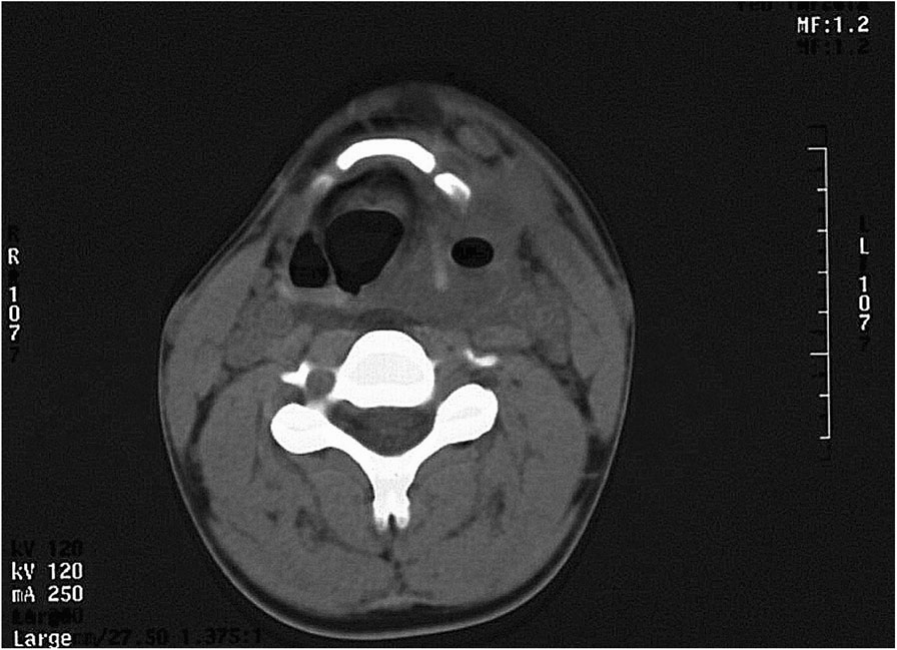
A CT image. Air pocket within the swelling tissues of the *left* neck

A 19-year-old man presented to the emergency room at 4:00 p.m. with a severe, recurrent left-sided neck abscess that manifested itself over the previous week. The patient had a high fever, ranging from 39.3–40.0 °C with left-sided odynophagia. A fluctuant neck mass could be felt, and the temperature of the skin over the mass was higher than normal. A complete blood examination revealed a WBC count of 15.98 × 10^9^/L, D-dimer levels that reached 1.92 μg/mL, and levels of plasma fibrinogen at 6.96 g/L. Ultrasonography indicated swelling of the tissues in the left side of the neck, and computerized tomography (CT) of axial sections of the neck showed an air pocket on the left side of the pharynx (Fig. 1). The patient had presented with a similar condition 2 years ago and was managed by incision and drainage of the neck abscess. At 8:47 p.m., the patient was sent to the operating room for incision and drainage, and at 9:27 p.m. (just before the administration of general anesthesia), his blood pressure suddenly dropped to 80/50 mm Hg and his blood oxygen saturation continuously decreased to 75 %. The patient also became tachycardic and dysphoric. A tracheotomy was performed and a laryngoscope was inserted instantly, which showed that there was purulence with blood rushing into the pharyngeal cavity. The condition of the patient continued to deteriorate, and all rescue measures proved ineffectual. He was ultimately pronounced dead at 11:00 p.m.

### Autopsy findings

**Fig. 2:**
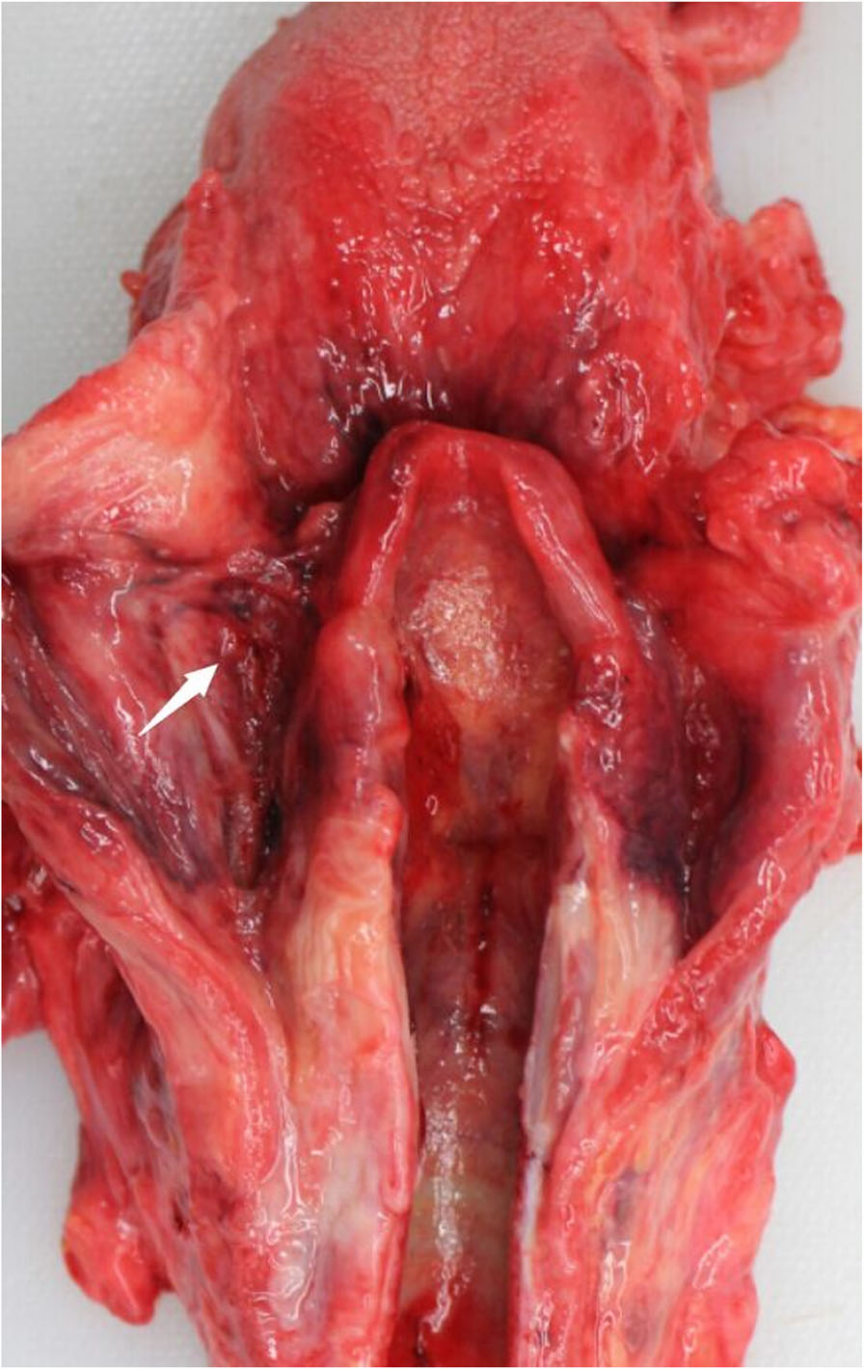
A sinus in the cranial end of the left-sided pyriform fossa (*white arrow*)

A forensic autopsy was performed on the 4th day after the patient’s death. A 5.0-cm-long vertical skin suture from the tracheotomy was observed in the middle of the anterior neck and a 3.0-cm-long horizontal and old surgical scar was observed above the skin suture. We found a 10.0 cm × 5.0 cm × 4.0-cm mass with suppurative necrosis and hemorrhage on the left side of the neck, and a 1.0-cm vertical tracheal incision was found under the isthmus of the thyroid gland. Hemorrhage and necrosis of the left lobe of the thyroid gland could also be observed. The cervical muscles were difficult to separate due to severe adhesion and inflammation of neck tissues, and remarkable swelling and a sinus were observed in the cranial end of the left-sided pyriform fossa (Fig. 2). Several small abscesses were also found on the root of the tongue. Brain edema was obvious and splinter subarachnoid hemorrhages could be seen externally. The heart weighed 315 g, and the thicknesses of the left and right ventricular walls were 1.3 and 0.3 cm, respectively. We additionally found some blood with purulence in the cavities of the trachea and bronchus. Edema was obvious in both lungs and numerous hemorrhagic spots could be found on the surface. No meaningful lesion was observed in other organs.

### Histopathologic and immunohistochemical findings

**Fig. 3:**
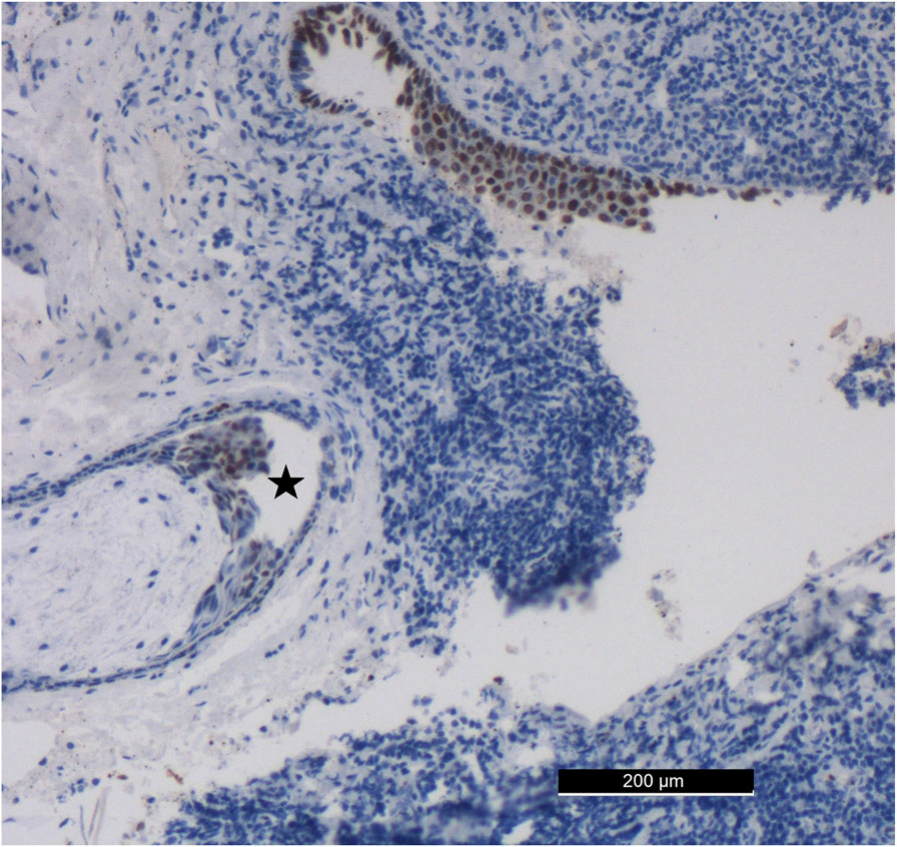
The P63-positive epithelial cells which surrounded the fistula (Five-pointed star) in the pyriform fossa sinus (IHC on P63 stain 100×)

**Fig. 4:**
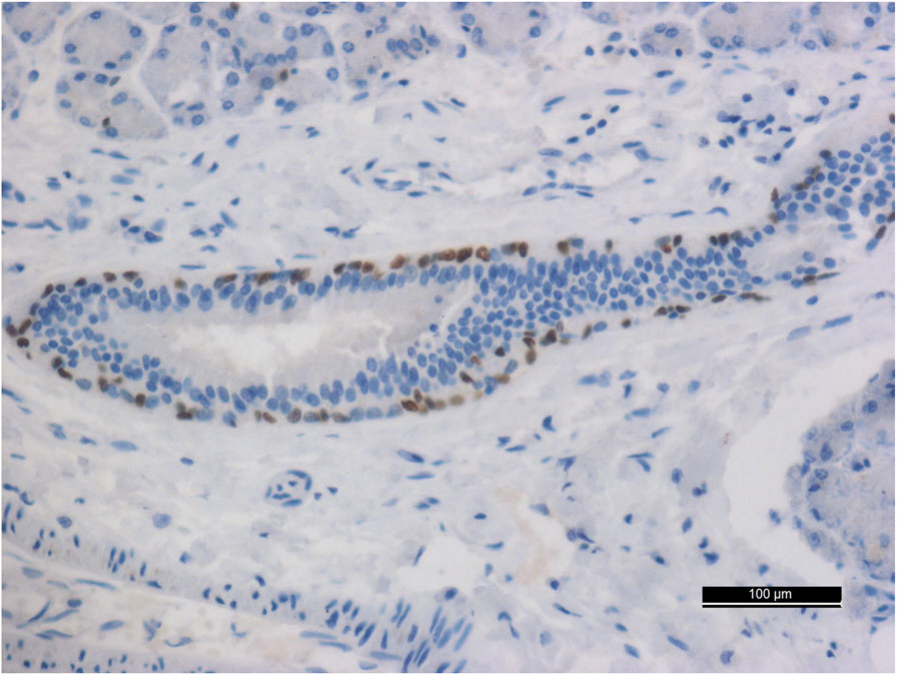
The fistulae structure surrounded by squamous epithelial cells in the neck tissue (IHC on P63 stain 200×)

**Fig. 5:**
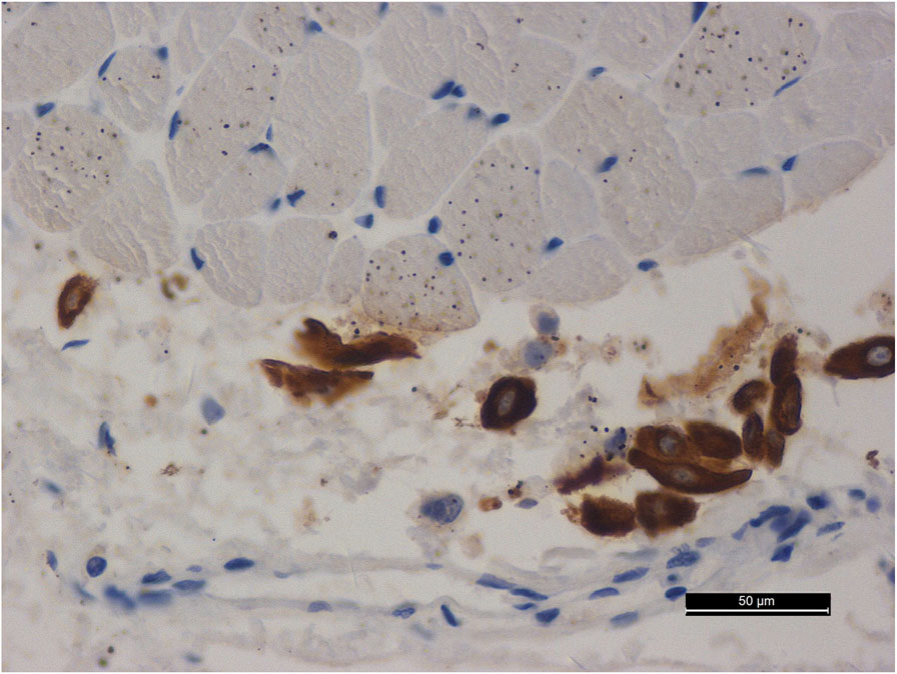
The PCK-positive epithelial cells in the neck tissue (IHC on PCK stain 400×)

We found hemorrhages and necrosis with numerous inflammatory cells within the tissues of the anterior left neck. Squamous epithelial cells with keratinizing or non-keratinizing cells and fibroplasia could be observed in the mesenchyme of the tissues in the left neck. Edema was severe in the submucosa of the left pyriform fossa, which contained numerous small abscesses. Multiple hemorrhages and necrosis with many inflammatory cells could be observed within the tissues of the tongue root. We found focal and scattered hemorrhages under the capsule of the left thyroid, and most of the acinar cells appeared atrophied. The mesenchyme of the left lobe of the thyroid became fibrotic with infiltration of numerous neutrophil granulocytes. Fibroplasia with infiltration of numerous inflammatory cells was also observed in the membrane of the left submandibular gland. Fistulas surrounded by squamous epithelial cells with keratinizing and/or non-keratinizing cells were found within the tissues of the pyriform fossa sinus. P63 was highly expressed in the squamous epithelial cells of the pyriform fossa sinus (Fig. 3) on IHC examinations. Squamous epithelial cells of variable maturity and fistular structures (which were positive for PCK and/or P63 on IHC), were distributed in the left cervical muscles (Figs. 4 and 5); and their nuclei were of different sizes. Numerous small abscesses could be observed in the sternocleidomastoid muscle. Some of the cytoplasm of the neck muscles was quite acidophilic and even appeared to be dissolving. Severe infiltration of lymphocytes and neutrophil granulocytes, and proliferation of fibers were observed in the mesenchyme. Microthrombi were found in many small vessels of vital organs such as brain, lungs, kidney and spleen; and the proportion of inflammatory cells in partial small vessels was high. Minor splinter hemorrhages were found in the myocardial mesenchyme of the interventricular septum. Emphysema and focal hemorrhage in some alveoli were also observed. In addition to edema and minor anemia, there were no significant pathologic changes in other organs; and toxicologic examination ruled out the presence of all common drug substances.

### Diagnosis

Postmortem findings and the neck CT manifestations (such as fistulas surrounded by squamous epithelial cells, severe neck swelling and thyroiditis; and the left-sided pyriform sinus and air bags) all conformed to the clinical presentations and pathologic changes typical of third bronchial fistula. The histopathologic examinations together with complete blood examinations revealed that the patient had a severe infection before death and the blood was in a hypercoagulable state. Thus, the cause of death was considered to be septic shock following a neck infection.

## Discussion

Branchial cleft anomalies are due to aberrant embryonic development, and can result from different malformations of the branchial apparatus. These may present as a fistula, sinus tract, or cyst, and encompass four known specific types [1]. First, branchial cleft anomalies are the rarest type, accounting for less than 1 % of the four types [9]; while second branchial cleft anomalies are the most common types, accounting for approximately 95 %. Third branchial anomalies are also rare and they account for 2–8 % of all such cases [3, 4, 10]. The prevalence of fourth cleft anomalies is low, at approximately 1–4 % [11]. Although all four types of branchial cleft anomalies are able to give rise to neck abscesses [1], they each have a unique anatomical appearance.

Pathologically, the presence of keratinizing and/or non-keratinizing squamous epithelial cells within neck tissue is thought to be a cytologic standard for diagnosing branchial fistulas [12]. According to the standard referenced above, IHC examinations of P63 and PCK in neck tissue are recommended for a further pathologic diagnosis. P63 is a specific protein that is expressed in the nucleus of squamous epithelial cells, and PCK is a specific marker of keratinizing epithelial cells expressed in the cellular cytoplasm. In the present study, we found the pyriform fossa sinus to be surrounded by P63-expressing squamous epithelial cells. Numerous fistular structures surrounded by positive P63-expressing squamous epithelial cells and PCK-expressing keratinizing epithelial cells were also observed in the left cervical muscles.

The pathway taken by fourth branchial cleft fistulas has been described as being similar to that of third branchial cleft fistulas. This makes it difficult to differentiate the two types of branchial anomalies in clinical practice; and they are grouped as pyriform fossa sinus tracts by some authors [5, 13]. A pyriform fossa sinus is most typically investigated with a CT scan [14], and the manifestation of air within a left neck swelling is considered to be a characteristic finding of a lesion in a third and/or fourth branchial fistula [15]. In a recent study on branchial anomalies, recurrent neck abscesses and suppurative thyroiditis are thought to be the primary clinical presentations of a pyriform fossa sinus [14]. In our case, the features shown by neck CT and the inherent clinical syndromes conformed to the characteristic presentation of pyriform fossa sinus.

Third and fourth branchial cleft fistulas occupy a fine distinction in anatomy, although the two types of branchial anomalies have almost the same clinical presentation. Studies have shown that the internal opening of third branchial cleft fistulas is located at the cranial end of the pyriform fossa, and the tract passes above the superior laryngeal nerve; while the fourth branchial cleft fistulas originate at the caudal end of the pyriform fossa and pass through the cricothyroid membrane beneath the superior laryngeal nerve [5, 16]. In our case, due to the patient’s operation 2 years ago and cryopreservation of the body, it was impossible to separate the tract and identify the pathway. However, the opening sinus in the cranial end of the pyriform fossa indicated that it was a third branchial cleft fistula. In a study of congenital branchial cleft anomalies, the recurrent rate of lateral neck abscess and suppurative thyroiditis induced by third branchial fistulas was reported to approach 42 and 45 %, respectively [5]. Open-neck surgery with excision of the fistular tract (preceded by the administration of appropriate antibiotics), is most commonly performed to control neck infection [7, 17]. In a study conducted by Nicoucar et al. [7], endoscopic cauterization treatment was recommended to effect a radical cure (with limited complications) for third branchial cleft fistulas.

Branchial cleft fistulas are not associated with high mortality, as we found few fatal cases using PubMed, Google Scholar or other mainstream publication search engines. However, according to our findings in the present case, such a fistula becomes a potentially lethal disease if misdiagnosis or delayed/faulty treatment occurs. Collection of the neck CT scans is particularly valuable for observing the fistular tract, sinus, or infection [14]. Histopathologic examination of neck tissue is critical for a correct diagnosis, and is considered to be the gold standard for branchial cleft fistulas [12]. IHC can aid in confirming the common pathologic presentations, such as fistulas surrounded by squamous epithelial cells in cervical muscles and other neck organs [1, 12]. In contrast, the fistular tract pathway should be confirmed because it is a key point in the diagnosis of the specific type of branchial cleft anomaly. The examination of the position of the fistular opening is a convenient method for a tentative diagnosis, and radiography of the fistula can provide a view of the pathway taken by the fistular tract. In practice, medical examiners should be made aware of this condition at autopsy if neck cysts or abscesses are found, as such information would be important in the diagnosis of the cause of death [18].

## Conclusions

This present analysis revealed that a proper histopathologic examination of the neck tissue along with a review of the medical history and examinations of CT and ultrasonographic images can provide a rapid and accurate diagnosis of third branchial cleft fistula. This commonly encountered and usually non-lethal disease can, however, potentially lead to death if the neck infection is not properly treated. In medico-legal practice, medical examiners need to make themselves aware of this condition, as this would be important in the diagnosis of the cause of death.

## Abbreviations

CT: Computed tomography
IHC: Immunohistochemistry
